# Tau Structures

**DOI:** 10.3389/fnagi.2016.00262

**Published:** 2016-11-08

**Authors:** Jesus Avila, Juan S. Jiménez, Carmen L. Sayas, Marta Bolós, Juan C. Zabala, Germán Rivas, Felix Hernández

**Affiliations:** ^1^Centro de Biología Molecular Severo Ochoa (Consejo Superior de Investigaciones Científicas-UAM)Madrid, Spain; ^2^Centro de Investigación Biomédica en Red de Enfermedades NeurodegenerativasMadrid, Spain; ^3^Departamento de Química Física Aplicada, Universidad Autónoma de MadridMadrid, Spain; ^4^Centre for Biomedical Research of the Canary Islands, Institute for Biomedical Technologies, University of La LagunaTenerife, Spain; ^5^Departamento de Biología Molecular, Facultad de Medicina, IDIVAL-Universidad de CantabriaSantander, Spain; ^6^Centro de Investigaciones Biológicas, Consejo Superior de Investigaciones CientíficasMadrid, Spain

**Keywords:** motifs, tau residues, posttranslational modifications, PHFs, tubuling binding, aggregation, microtubule-associated proteins

## Abstract

Tau is a microtubule-associated protein that plays an important role in axonal stabilization, neuronal development, and neuronal polarity. In this review, we focus on the primary, secondary, tertiary, and quaternary tau structures. We describe the structure of tau from its specific residues until its conformation in dimers, oligomers, and larger polymers in physiological and pathological situations.

Tau is a neuronal protein that was found (Weingarten et al., 1975) associated to microtubules, together with other microtubule-associated proteins (see Matus, 1988 for a review). Tau protein plays a role in microtubule assembly and dynamics that may regulate neuron morphology (Drubin and Kirschner, 1986; Caceres and Kosik, 1990; Panda et al., 1999). The roles of tau in axon development and navigation (Dawson et al., 2001; Sayas et al., 2015), dendritic spine function (Ittner et al., 2010), or long-term depression (Takashima, 2012), have been described.

Tau protein(s) is (are) encoded by a single gene, *mapt*, that is located on chromosome 17 in humans (Neve et al., 1986). This gene is transcribed into a nuclear RNA that yields six different isoforms, lacking or containing exons 2, 3, and 10 by alternative splicing (Avila et al., 2004; Andreadis, 2006). Exon 10 contains a microtubule-binding region similar, but not identical, to other three additional microtubule-binding regions. Thus, isoforms containing exon 10, resulting in tau with four microtubule (tubulin)-binding regions (repeats) are known as tau 4R, whereas alternative spliced isoforms, lacking exon 10, are known as tau 3R.

## Tau primary structure

As previously indicated, there are mainly six tau isoforms in brain [and central nervous system (CNS)], showing differences in their primary structure due to the presence or absence of some specific exons. In addition, in peripheral nervous system there are additional tau isoforms containing an extra exon, exon 4a (Goedert et al., 1992). However, the longest human CNS tau isoform with 441 residues has usually been chosen as a model to exemplify tau primary structure (Goedert et al., 1989). That isoform contains exons 1, 2, 3, 4, 5, 7, 9, 10, 11, 12, and 13. Within those 441 residues there is a high proportion (*80*) of phosphorylable residues (serine and threonine) and a low proportion, compared to other proteins, of hydrophobic aminoacids [alanine (*34*), valine (*25*), isoleucine (*15*), leucine (*20*), methionine (5), phenylalanine (3), tyrosine (*5*) or tryptophan (*0*)].

Phylogenetic analyses of proteins with tau-like microtubule-binding regions have indicated the existence of a protein family of microtubule-associated proteins composed by tau, MAP2, and MAP4 proteins. Also, similarities with some proteins from invertebrate organisms and with a histone acetyltransferase subunit (KANSL1), have been recently reported (Sündermann et al., 2016).

Comparison of sequences of tau proteins from different origins has indicated a larger variability at the N-terminal half of tau than at C-terminal tau (Leon-Espinosa et al., 2013). Interestingly, the localization of threonine (or alanine) residues do not present a random distribution and they are present in a higher proportion at the N-terminal region of tau protein (Avila et al., 2012). Regarding the possible function of exons 2 and 3, it has been described that they might play a role in the association of tau protein with different cell membrane proteins like apolipoprotein ApoA1 (preferentially binding to tau with those exons) or synaptophysin (preferentially binding tau without those exons; Liu C. et al., 2016). Curiously, a binding site for NRF2, a transcription factor involved in antioxidant responses, is present in the first intron of the *mapt* gene and it may facilitate exon 3 inclusion in tau protein, an inclusion that could have a protective role (Wang et al., 2016).

Another important feature related to the primary structure of tau protein is the presence of different types of posttranslational modifications (PTMs) of specific residues. Among these modifications are phosphorylation, acetylation, deamidation, methylation, O-Glycylation, or ubiquitination (Avila et al., 2004; Avila, 2009; Morris et al., 2015; Huang et al., 2016; Iqbal et al., 2016). Furthermore, loss of N-terminal or C-terminal regions by truncation are very well-known (García-Sierra et al., 2008; Avila et al., 2015).

Although tau protein has been considered as an intrinsically disordered protein (IDP), modifications by phosphorylation or other posttranslational modifications could facilitate a “gain” in secondary structure that could facilitate the appearance of α-helix or β-sheet regions (see below).

### Focus in specific tau residues

**Figure 1:**
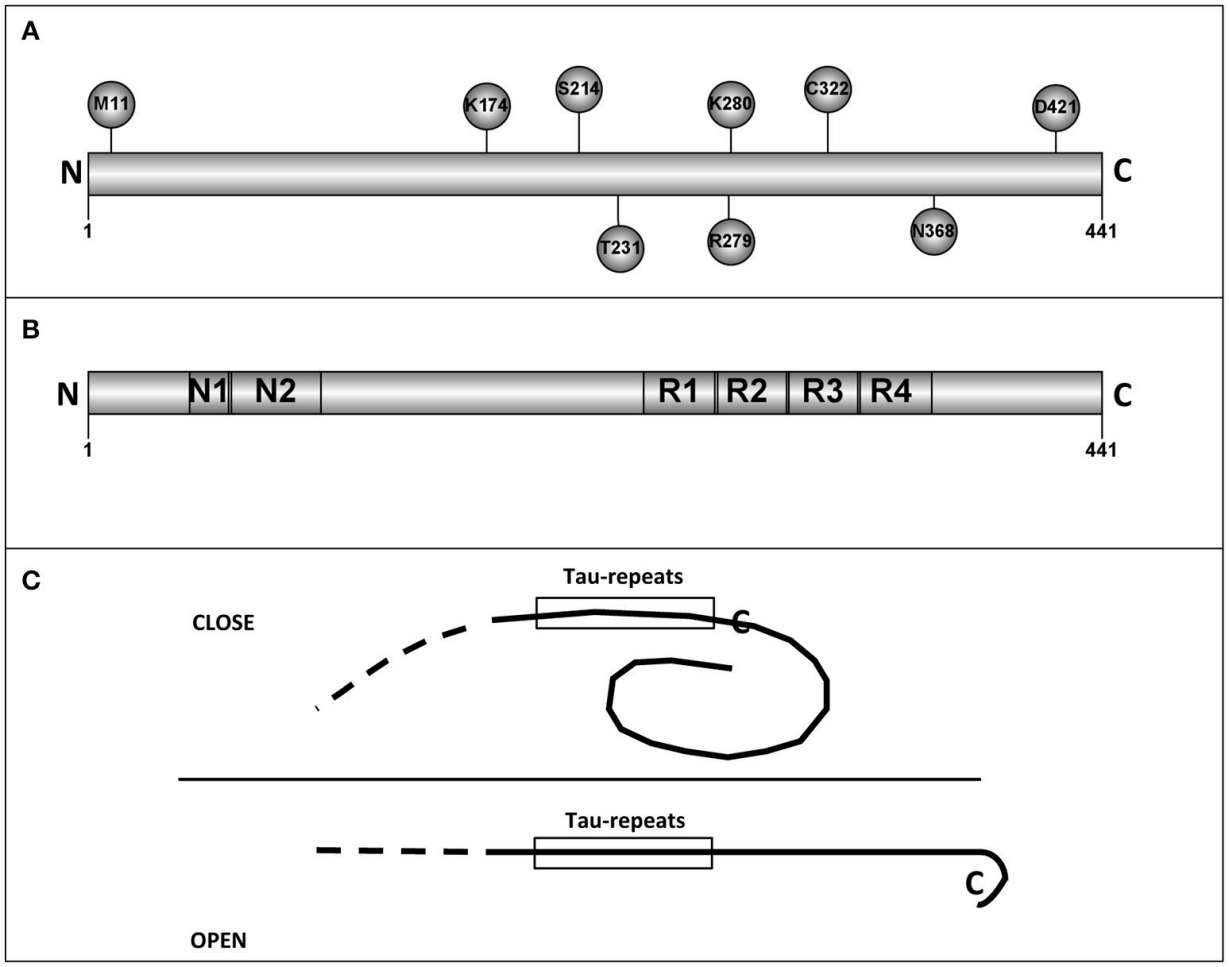
**Tau residues and tau regions. (A)** The localization in tau molecules of some tau residues is shown. **(B)** The different tau regions, including the microtubule binding repeats are shown. **(C)** Intramolecular interactions, like that described for “paper-clip” structure (see text and reference, Jeganathan et al., 2006) could prevent the tau self-interaction by masking the region(s) involved in that interaction.

As previously mentioned, primary structure of tau protein can be divided into two regions, N-terminal, and C-terminal regions. The C-terminal part shows less variability among tau proteins from different species. This fact may suggest that some of these conserved residues could be essential for some tau functions. Hence, related to tau functions, we have selected 11 of the conserved residues that could be important for human tau's role. Only three of them are at the N-terminal half of the protein. The chosen residues are: M11, A152, K174, S214, T231, R279, K280, C322, N368, S409, and D421 (Figure 1A shows some of them).

Methionine 11 could be the first residue for a tau truncated protein, in the absence of the first methionine. Tau protein starting at M11, has a decreased interaction with Alz50 antibody suggesting possible changes in the putative tau tertiary structure (Carmel et al., 1996; see below).

Lysine 174 can be acetylated. This modification results in a toxic protein (Min et al., 2015). Serine 214 can be phosphorylated by protein kinase A (Scott et al., 1993) and it has been suggested that this modification might play a positive role in the binding of tau to microtubules (García de Ancos et al., 1993). Threonine 231 is followed by two proline residues. T231 is modified by different proline directed kinases like GSK3, JNK, MAPK or cdk5 (Illenberger et al., 1998; Reynolds et al., 2000). As indicated, T231 can be phosphorylated and the following prolines can have a cis or trans conformation which could regulate the posterior dephosphorylation of P-T231 (Lu et al., 1999). This is an important task since the presence of this phosphoprotein could be toxic (Nakamura et al., 2012). Also, P-T231 is present in peripheral nervous system in submandibular gland and, curiously, the level of phosphorylation decreases with increasing Braak stage of Alzheimer disease (Dugger et al., 2016). Tau is mainly an axonal protein. However, in Alzheimer disease and other tauopathies it is also present at dendritic spines where may play a toxic role. This toxic role could be the consequence of tau modifications like phosphorylation, truncation, or acetylation. The knowledge of the role of acetylated tau in tauopathies is increasing gradually. For example, in the work of Tracy et al. is described, in an elegant way, a possible mechanism explaining the role of acetylated tau (at lysines 274 and 281). In this article, memory impairment is studied by using a mouse model which express human tau with lysine to glutamine mutations to mimic lysine acetylation (Tracy et al., 2016). Acetylated tau reduces the levels of kidney and brain expressed protein (KIBRA) present at synapses and this may produce a decrease in synaptic efficiency (Tracy et al., 2016). More recently, it has been shown that acetylated tau (at lysines 274 and 281) destabilizes the cytoskeleton in the axon initial segment (Sohn et al., 2016).

Arginine 279 can be deaminated (Dan et al., 2013) and deamination results in a reduced ability of modified tau to bind to microtubules (Dan et al., 2013) and perhaps it could facilitate its self-aggregation (Montejo de Garcini et al., 1986).

Lysine 280 loss facilitates tau self-assembly and exacerbates tau toxicity (Gorsky et al., 2016; Wang and Mandelkow, 2016).

Cysteine 322 is modified by oxidation, a modification that regulates tau self-assembly (Kim et al., 2015; Wang and Mandelkow, 2016).

Cleavage of asparagine 368 by an asparaginase results in the appearance of a very toxic fragment (Zhang et al., 2014).

Serine 409 is a good substrate for Rho kinase and other related kinases (Amano et al., 2003). Recently, it has been suggested that ROCK phosphorylates tau protein decreasing its further degradation (mainly for Tau4R). This may result in tau accumulation, as occurs in some tauopathies like corticobasal degeneration or progressive supranuclear palsy. As a results, the use of ROCK inhibitors has been suggested for the treatment of those diseases (Gentry et al., 2016).

Finally, truncation of aspartic 421 facilitates tau aggregation and toxicity (García-Sierra et al., 2008) and phosphorylation at serine 422 could enhance SDS-stable dimer formation (Tiernan et al., 2016).

Other residues could be relevant because they can be modified by phosphorylation (see review by Hanger et al., 2009) or because their mutations could facilitate the development of some tauopathies (for a review see Goedert and Jakes, 2005).

A recent example of aminoacid changes resulting in toxic function is the mutation of alanine 152 to threonine which causes age-dependent neuronal dysfunction, possibly through a mechanism in which NMDA receptors could be involved (Decker et al., 2016; Maeda et al., 2016). The consequences of having A152T mutation are different than those due to the presence of other tau mutations. Commonly, tau mutations could facilitate the assembly of large tau polymers. However, A152T mutation promotes the formation of smaller toxic tau oligomers. In this way, using a *C. elegans* tauopathy model, expressing A152T tau mutant, it was found a reduced lifespan and severe locomotor deficits. This pathology was due to the presence of the indicated toxic tau oligomers but not to that of insoluble aggregates (Pir et al., 2016).

### Tau regions or motifs involved in the binding of tau to other proteins

#### Tau, α-tubulin binding protein

The development of the mammalian CNS requires the generation, migration, and differentiation of neurons. Each of these cellular events needs a highly dynamic microtubule cytoskeleton. It is necessary for, among others, the separation of chromatids in mitosis, nuclear translocation during migration, and the outgrowth of neurites. Microtubules are polymers composed of globular subunits of α- and β-tubulin, two members of one of the largest families of genes and proteins (Turk et al., 2015). These two families of tubulins, with an approximate molecular mass of 50 kDa, are GTP-binding proteins and form an αβ-tubulin heterodimer which represents the structural and functional unit of the microtubule. It has been thought for a long time that the GTP-bound to the α subunit (non-exchangeable) is never hydrolyzed being inaccessible for biochemical manipulations, while those bound to the β subunit (exchangeable) are hydrolyzed twice. First, during the formation of the dimer and second, after microtubule incorporation. The process of tubulin folding follows a sophisticated pathway in which several molecular chaperones are involved. α and β subunits are folded independently with the GTP bound to each one. Among all the chaperones involved, including CCT and prefoldin, tubulin folding cofactors are molecular chaperones specifically required for αβ-tubulin dimer formation and the acquisition of its quaternary structure (Serna and Zabala, 2016).

On the other hand, as adult neurons are not dividing cells, they need to stabilize the microtubule cytoskeleton in order to maintain neuronal processes such as the fast axonal transport through long distances. Tau was identified (1–3) as a microtubule associated protein mainly expressed in neurons. The main role of tau is the stabilization of the axonal microtubules contributing to decrease their intrinsic dynamic instability. Furthermore, tau maturation implies a fine regulation of its expression in order to produce appropriate amounts of different isoforms by alternative splicing. This is because the isoforms regulate differentially microtubule dynamics (Amos, 2004). Paclitaxel is considered a mitotic inhibitor for its ability to stabilize microtubules suppressing their dynamic, which is a requirement for a proper cell division. For this reason, it is widely used in cancer treatment. Extensive studies have been directed to the precise understanding of the interaction site in the β-tubulin subunit. Paclitaxel and different microtubule-stabilizing compounds such as epothilones compete at the same general site for binding to microtubules, although paclitaxel does not bind to yeast microtubules, for they share overlapping but not identical binding sites. This difference was found to reside in few amino acid changes in the β-tubulin binding region (Gupta et al., 2003). As described above, tau is an intrinsically disordered and extended polypeptide that contains a microtubule binding domain with three or four binding regions that allow binding to several heterodimers at the microtubule lattice (Figure 1B). These tubulin-binding regions are characterized by the presence of prolines as well as basic residues to counteract the presence of acidic residues at the C-terminus existing on the surface of microtubules. It seems that binding to microtubules in different heterodimers favors the stabilization of the microtubule and such binding occurs in the same site where paclitaxel binds to mammalian microtubules. Paclitaxel and discodermolide, among others, compete with tau for binding the β-tubulin. In fact, in polymerization experiments performed in the presence of such kind of compounds, pelleted microtubules contain less tau polypeptides. In addition, the sequence THVPGGN contained in the tau binding domain is similar to the α-tubulin sequence TVVPGGDL, present in its extended loop (Rowinsky and Donehower, 1995). When for different reasons tau is detached from microtubules, they become destabilized and also due to the fact that tau is a disorganized protein with interacting surfaces exposed, it tends to aggregate forming PHFs. This situation led to the formation of neurofibrillary tangles (see below) and finally to neuron death. In this case, induction of tau degradation might be an important therapeutic approach. Chu et al. (2016) have recently developed peptides derived from α- and β-tubulins that recognized tau proteins connected to peptides recognized by E3 ubiquitin ligases and also to a cell penetrating peptide. They found that some of these peptides induce tau poly-ubiquitination and as a consequence its degradation in an AD mouse model decreasing cytotoxicity (Chu et al., 2016).

#### Other tau binding proteins

Actin or protein phosphatase 2A binds to tau, through tau repeats (Correas et al., 1990; Bakota and Brandt, 2016). On the other hand, tau motifs like PXXP may interact with membrane proteins containing SH3 domains (Hwang et al., 1996) or with other proteins, like Fyn tyrosine kinase that can be also localized at the cell membrane (Lau et al., 2016). Tau, N-terminal region, could be involved in the interaction with annexin A2 (Bakota and Brandt, 2016).

The presence of exon 3 facilitates the binding of other proteins such as ApoE, 14-3-3 zeta or synaptotagmin (Liu C. et al., 2016). Also, tau interacts with alpha synuclein, probably through the basic region containing residues 166–226 (Oikawa et al., 2016). As mentioned above, tau interacts with other microtubular proteins, such as the EB1 and EB3 proteins (7), in developing neuronal cells. Characterization of tau binding to EBs is currently under study. Also, tau can interact with other proteins, like the RNA binding protein TIA1, through a RNA intermediate (Vanderweyde et al., 2016). More recently, the interaction of tau with other proteins like ferritin and transferrin has been reported (Jahshan et al., 2016). Also, the residues 139–143 could be involved in the binding to heparin and the residues 336–343 and 347–351 could be involved in chaperone mediated autophagy (Wang et al., 2009). By using a protein-protein mining tool, the laboratory of Dr. Li Weil in China (http://liweilab.genetics.ac.cn/tm/gene.php?st=gn&gn=mapt&gi=4137&ti=9606) have described 172 genes related to tau gene.

## Tau secondary structure

Tau protein in solution can be considered as an intrinsically disorder protein (IDP). It means that, as probed by different techniques including circular dichroism (CD), Fourier transform infrared spectroscopy (FTIR), X ray diffraction, fluorescence, and some other methods, this protein behaves as a kind of random coil, lacking a well-defined secondary structure (for a full review on intrinsically disordered proteins, IDPs, see reference Skrabana et al., 2006). Tau is a highly soluble and heat-stable protein, in agreement with the lack of a well-defined secondary and indeed tertiary structure. The first report on tau protein structure (Cleveland et al., 1977) appeared 9 years before PHFs were reported to be composed mainly of this protein (Grundke-Iqbal et al., 1986a; Kosik et al., 1986; Nukina and Ihara, 1986). The CD spectrum reported by Cleveland (Cleveland et al., 1977) did not show any relevant secondary structure in a significant extent. This lack of structure was confirmed later by Woody et al. (1983) from proton NMR studies of microtubule- associated proteins. A large number of proteins contain, along their sequence, apparently unordered segments that, on the other hand, possess a clearly defined function once they face their interacting partners. This seems to be the particular case of the so-called *conformational diseases*. Aggregation of totally or partially disordered proteins has been repeatedly reported to be associated with many human neurodegenerative diseases, including Huntington's, Creutzfeldt-Jakob's, Parkinson's, as well as Alzheimer's disease, and other neurodegenerative disorders as the so-called *tauopathies* in the case of tau (Avila, 2006). However, in most cases, and the tau protein is one of them, the molecular mechanism of aggregation and even the fine structure of the final aggregate—as it is the case for the PHFs formed by tau protein—remains obscure and controversial.

Research on the secondary structure of tau protein is closely tightened to that concerning the detailed molecular structure of PHFs. Some months after the publication of the reports confirming that tau protein was the main component of PHFs, filaments resembling the PHF structure were obtained “*in vitro*” from tau protein purified from porcine brain (Montejo de Garcini et al., 1986). Thereafter, the door was open to study the mechanism of tau aggregation leading to PHFs formation and the subsequent search for the inhibition of the aggregation process as a therapeutic target. However, obtaining PHFs from tau protein resulted to be a not so easy task, and it seemed that particular phosphorylation sites on tau had to be phosphorylated (Biernat et al., 1992; Wille et al., 1992). Most intriguing was to reconcile the subtle helical structure found in PHFs with a tau protein devoid of any relevant secondary structure, as deduced from CD and FTIR (Schweers et al., 1994). On the other hand, the detailed molecular structure of PHFs has remained controversial and at the same time no well-defined structure has been reported for tau protein. Already in 1986, before tau was shown to be the main component of PHFs, the characteristic reflection at 4.7 Angstrom, corresponding to a β-pleated sheet structure, was reported to be found after X-ray diffraction on PHFs (Kirschner et al., 1986). However, 8 years later, similarly to tau protein, PHFs were reported to be devoid of any secondary structure, as deduced from X-ray diffraction (Schweers et al., 1994). The presence of β-amyloid in the PHFs preparation was considered as a plausible explanation for the 4.7 Angstrom reflection reported previously. Nevertheless, the same group reported later circular dichroism and FTIR data of a tau derived peptide confirming a pronounced β-structure on assembling. Their data indicated that PHF assembly was dependent of a local sequence in tau protein, at the beginning of the third repeat, forming β-structure (von Bergen et al., 2000). Also, based on X-ray diffraction data, there was a claim that peptides derived from tau protein—but not the full-length protein—formed cross-β structure (Giannetti et al., 2000). On the other hand, evidences for α-helix structure were also reported. Based on CD and FTIR data, Sadqi et al. claimed that PHFs isolated from brain samples of AD patients contained a high proportion of α-helices (Sadqi et al., 2002). A percentage of α-helix higher than β-sheet or unordered structure has also been found for PHFs suspended in Tris buffer (Goux, 2002). A tau fragment corresponding to the microtubule-binding region of the three repeat tau isoforms, as probed by high resolution NMR, has revealed three regions involved in microtubules binding, exhibiting a high preference for α-helix. A β-strand in a stretch between two α-helix segments, alternatively, is involved in the aggregation process to form PHFs (Eliezer et al., 2005). The authors conclude that the cross-β structure might be limited to short stretches at the core of the PHF structure, while α-helices would be found within the core of the microtubule-binding domain, as well as at the N- and C-terminal regions of tau, therefore explaining the high content of α-helices reported (Goux, 2002; Sadqi et al., 2002; Eliezer et al., 2005). This would be in agreement with the observations of Kunjithapatham et al. that proneness of tau to aggregate into α-helical structures could be a consequence of its microtubule-binding capacity (Kunjithapatham et al., 2005).

One of the main drawbacks to study tau protein structure comes from its flexible and intrinsically disordered nature. This type of proteins, offering a large population of conformations, is not amenable to be studied by crystallographic methods. Therefore, spectroscopy, particularly NMR, as well as CD and Fourier Transform Infrared have been the most relevant sources of structural information. The paper by Mukrasch et al. (2009) is probably the most complete description of the full secondary structure of the longest, 441 amino acid residues, and form of tau protein. The NMR data reveal that 343 out of 441 amino acids comprising tau are devoid of any ordered structure. Only small segments of the sequence present transient elements of secondary structure: six segments display propensity to form β-strands, three segments show poly-Pro helices and, finally two segments composed of ten amino acids, 114–123 and 428–437, within the N-terminal projection domain and the C-terminal domain present transient α-helix structure (Mukrasch et al., 2009; Wang and Mandelkow, 2016). No relevant sequence homology has been found for the 428–437 segment displaying α-helix propensity. On the other hand the α-helix segment at the projection domain 114–123 presents homology with an α-helix segment on cytochrome B5 (Qian et al., 2001). Most interesting is the finding that tau protein, in agreement with previous reports, presents a highly dynamic structure with many compact conformations and a complex network of long range transient contacts. This description is far from the elongated molecule originally described from electron microscopy (Wille et al., 1992), and is closer to the hairpin structure in which both N- and C-terminals contacted with each other (Jeganathan et al., 2006).

Tau protein has a large number of potential sites of phosphorylation (Grundke-Iqbal et al., 1986b; Bancher et al., 1989; Wang and Mandelkow, 2016), 80 Ser or Thr and 5 Tyr. The phosphorylation state of the protein is developmentally regulated and closely related to its function. Phosphorylation is involved in regulating the interaction with different partners as microtubules and nucleic acids (Camero et al., 2014; Multhaup et al., 2015), in the protein location and, indeed it is related to the PHF and tangles formation (Wang and Mandelkow, 2016). In fact one of the initial observations related to the abnormal tau aggregation into PHFs was that tau within this complexes is abnormally hyper-phosphorylated (Grundke-Iqbal et al., 1986b). Tau protein is a dynamic structure displaying a complex network of intramolecular long-range interactions, lacking a well-defined secondary structure -except for a number of short segments displaying some transient proneness to acquire particular structural elements. On the other hand, phosphorylation regulates all tau functions: development, spreading and location, partners interaction and of course PHFs formation and evolution, leading therefore to the question: how does phosphorylation affect the secondary structure of tau? After some mutations or serine and threonine residues in tau into glutamic acid, Bibow et al. have suggested that phosphorylation might induce the opening of the transient folded structure of tau (Bibow et al., 2011). Finally, NMR experiments carried out on a peptide derived from tau protein have shown how phosphorylation stabilizes the α-helix structure on tau (Sibille et al., 2012), therefore suggesting the possibility of a higher content of α-helices in the hyper-phosphorylated tau building PHFs.

## Tau tertiary structure

Electron microscopy analysis done by Hirokawa's group (Hirokawa et al., 1988) suggested that tau could have an extended fibrillar form. However, by looking at the epitopes recognized by the antibody Alz50, it was found that such antibody reacts with residues present at the N- and C-terminal regions, suggesting a possible tertiary structure in tau protein (Carmel et al., 1996). Although, nuclear magnetic resonance and small-angle X-ray scattering indicate that tau is an intrinsically disordered protein (Qi et al., 2015), a “paper-clip” structure has been suggested for some molecules of tau monomers. This model was obtained by FRET measurements on tryptophan mutants of tau protein (Jeganathan et al., 2006). The presence of a “paper-clip” structure may suggest the presence of intramolecular interactions between, at least, two different regions of tau molecule. If one of those regions is involved in tau self-interaction, the opening of the “paper-clip” structure could facilitate that self-aggregation (see Figure 1C). As indicated, tau is an intrinsically disordered protein and methods to determine its tertiary structure like X-ray crystallography could not be suitable to describe that structure and nuclear magnetic resonance (NMR) studies could be advisable to do it (Lippens et al., 2016).

NMR spectroscopy of isolated tau have shown some conformational changes upon phosphorylation of tau at its threonine 231, suggesting the stabilization of a small α-helix at that region (Sibille et al., 2012); or upon phosphorylation at the Ser 202 and Thr 205, a modification that could be recognized by AT8 antibody. Thus, modification of those sites could result in an unusual turn conformation (Gandhi et al., 2015).

## Tau quaternary structure

Tau protein could form dimers, oligomers and larger polymers. In the formation of tau dimers or oligomers, cysteine residues may play an important role (Takashima, 2013; Soeda et al., 2015). These cysteine residues are present at the microtubule-binding domain (MTBD) of tau protein that is also involved in tau-tau interaction. Nevertheless, for the formation of tau aggregates or fibers, it is not essential the formation of intramolecular cysteine_cysteine links, and the formation of tau fibers could be facilitated by the presence of polyanions like heparin (Huvent et al., 2014). It is discussed if tau dimers could be toxic (Cowan and Mudher, 2013), but there is some evidence indicating that tau trimers could be the toxic species (Tian et al., 2013). Larger granular tau oligomers, composed by around 36 tau molecules have been also described as possible toxic agents (Maeda et al., 2007). These oligomers may precede the formation of tau filaments found in different tauopathies. In Alzheimer disease, mainly two types of tau filaments have been described: paired helical (PHF) or straight (SF) filaments. By doing “*in vitro*” analysis, it has been proposed that a difference between PHFs and SFs could be the presence, in PHFs, of sulfated glycosaminoglycans, like heparan sulfate or chondroitin sulfate, associated to tau polymers (Arrasate et al., 1997). On the other hand, highly sulfated, heparan sulfate (heparin) facilitates tau aggregation to form filaments (Goedert et al., 1996; Pérez et al., 1996). In addition, glycation of tau filaments results in the formation of larger aggregates similar to neurofibrillary tangle aggregates (Ledesma et al., 1996).

Tau aggregates and fibrils could have a role in tau spreading. Tau spreading has been a hot topic within recent years (Clavaguera et al., 2009; Sanders et al., 2014; Peeraer et al., 2015). However, since this review is focused on the structure and not in the (toxic) function of tau aggregates, we enclosed reference (Wang and Mandelkow, 2016) for a good review of that topic.

**Figure 2:**
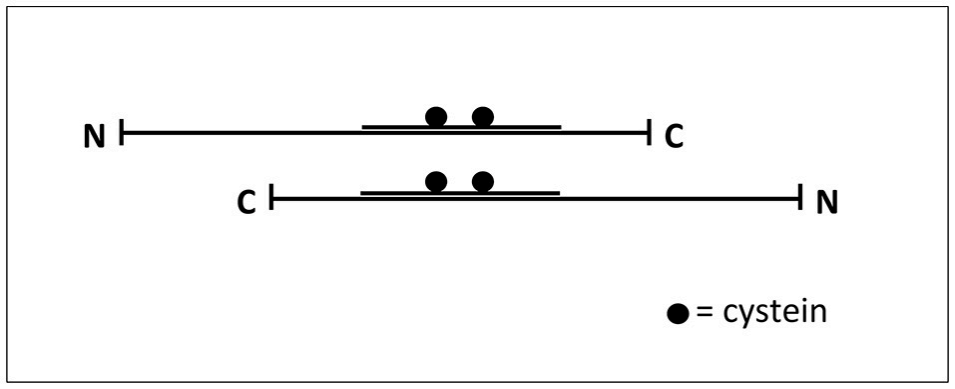
**Interaction tau-tau**. Tau protein could be found as an extended common long protein that could bind to other proteins, including itself, in this extended fashion (Hirokawa et al., 1988). The Figure shows how two tau molecules could interact, in an antiparallel fashion, through their microtubule-binding domain where cysteine residues are present.

It has been described that tau filaments are assembled by the self-association of tau molecules (Montejo de Garcini et al., 1986), in an anti-parallel arrangement, through the MTBDs. That arrangement could facilitate the interaction through cysteine and other residues present in that domain (Ksiezak-Reding and Yen, 1991; see Figure 2). Tau fragments containing the MTBD could also assemble into fibrillar polymers (Hirokawa et al., 1988; Jeganathan et al., 2006) and even a very small fragment composed by the hexapeptide VQIVYK, present in the MTBD, is able to form filaments (von Bergen et al., 2000).

On the other hand, when tau molecules are bound to microtubules, the MTBD is not accessible to facilitate tau-tau association. However, it has been reported that tau, bound to microtubules, could form dimers through its N-terminal region (Feinstein et al., 2016). These regions and also, perhaps, the C-terminal region could play a role in the spatial organization of axonal microtubules, keeping parallel microtubules despite of macromolecular crowding (Méphon-Gaspard et al., 2016).

Although mainly fibrillar polymers could be obtained “*in vitro*,” using only pure tau proteins, the assembly of the protein could be facilitated upon addition of anionic compounds (see for example Avila et al., 2004, for a review). These fibrillary structures are dynamic polymers, as shown in cell culture models (Guo et al., 2016).

**Figure 3:**
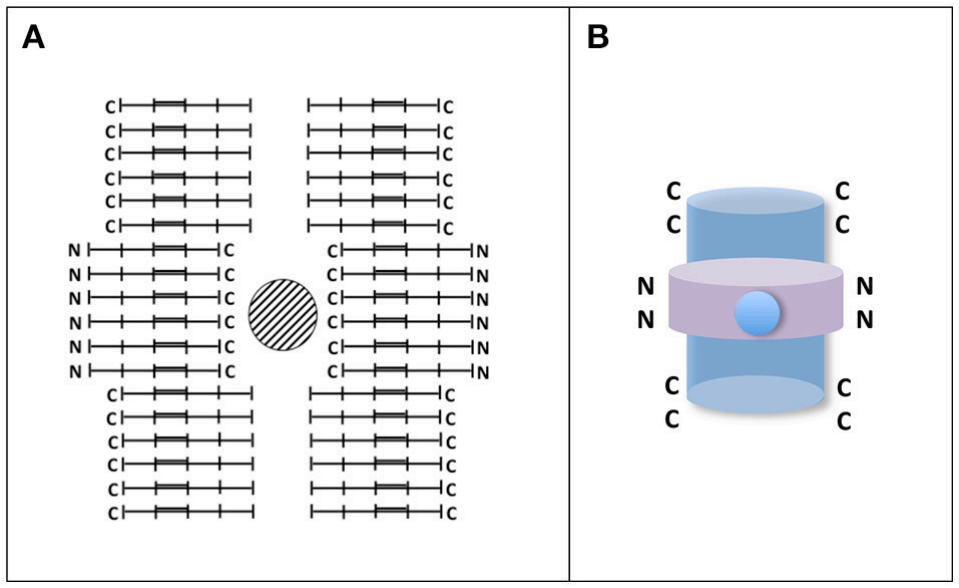
**Hypothetical tau circular-like structure. (A)** Tau granule (circular-like) structures have been previously reported (Rábano et al., 2013; Takashima, 2013). In the Figure, it is shown an hypothetical closed structure playing with parallel and antiparallel tau molecules. In that structure, a hole in the center surrounded by C-terminal sequences of tau molecule can be found. Since tau C-terminal sequences contain several acidic residues, it is possible the reaction of those residues with metals. It may take place in argylophylic grains. **(B)** A 3D-like image of the structure is shown. In that image parallel and antiparallel forms, with the C-terminal (blue) or N-terminal (violet), regions of tau molecule at the surface, could be present. It is proposed that those having the C-terminal inside the structure could react with cations.

Apart from fibrillary structures there are other tau polymers showing different structures like Pick's bodies or argylophilic grains, among others (see for example Rábano et al., 2013). The assembly of those structures could be facilitated by the presence of other compounds or by differences in the level of tau phosphorylation. Indeed phospho-tau dephosphorylation by protein phosphatase 2A could switch the formation of fibrillary polymers to argylophilic gain-like morphology (Hu et al., 2016). Curiously, injection in mouse brain of brain extracts from patients with tauopathies showing different tau structures could reproduce “*in vivo*” the appearance, in mouse, of those structures (Clavaguera et al., 2013; Sanders et al., 2014). It suggests that some additional compounds, in addition to tau, could be involved in raising the final morphology of tau structures. Also, those different morphologies could be the consequence of different arrangements of tau molecules, which could be found either in parallel form, mixed of parallel and anti-parallel forms, or anti-parallel form (see Figure 3). For example the hypothetical structure shown in Figure 3 could be the result of assembly of parallel and antiparallel forms. Some of them could aggregate around a cationic (metal) structure, whereas other could form antiparallel aggregates. Also, recently, has been shown that different structures could arise from the mixture of full length tau and tau fragments (Ozcelik et al., 2016). Nevertheless, when the injection in mouse brain was done using only synthetic tau protein, mainly fibrillar polymers were obtained (Iba et al., 2013).

Although, in a general way, it has been suggested the transition from soluble monomeric tau protein to the formation of large, insoluble, tau aggregates (Takashima, 2013), more studies are needed to test if tau protein follows a protein phase transition in a similar way to that described for the formation of FUS protein aggregates in amyotrophic lateral sclerosis (Patel et al., 2015). Recent findings have established that a variety of intracellular membrane-less organelles and micro-compartments behave as liquid droplets inside the cell that are formed by liquid phase transitions from cytoplasm, suggesting that phase separation play an important role in the functional organization of the cell (Hyman and Simons, 2012). Intrinsic disordered proteins containing low complexity domains (LCD) prone to aggregate have been shown to be more susceptible to phase separate (Hyman and Simons, 2012; Aguzzi and Altmeyer, 2016). In this regard, the assembly of FUS into dynamic non-membrane compartments was found to be dependent on the prion-like domain of the protein, a type of LCD, which is thought to be involved in the formation of toxic aggregates (Patel et al., 2015). In fact, this domain is also present in tau protein and other aggregation-prone proteins associated to aging diseases. Time-dependent FUS aggregation led to the formation of disease-linked solid aggregates and aberrant liquid to solid phase transition was accelerated by ALS patient mutations, suggesting that the pathological aggregates could result from unregulated phase transitions linked to protein modifications that alter its assembly properties. Therefore, factors modulating the relative abundance of the different aggregation states of an amyloid forming protein [as glycosylation (Liu K. et al., 2016) or crowding (Wu et al., 2015) in the case of tau protein] may have a major impact in the formation of dynamic liquid micro-compartments and in the liquid-to-solid phase transition leading to pathological aggregation. The maintenance of the proper balance of physiological phase transitions may constitute a novel therapeutic strategy against neurodegeneration.

## Author contributions

All the authors contributed equally to the outline, drafting, and editing of the present manuscript.

## Funding

This work was supported by grants from the Spanish Ministry of Economics and Competitiveness, Plan Estatal de I+D+i.

### Conflict of interest statement

The authors declare that the research was conducted in the absence of any commercial or financial relationships that could be construed as a potential conflict of interest.
